# Antiobesity effect of *Kaempferia parviflora* accompanied by inhibition of lipogenesis and stimulation of lipolysis

**DOI:** 10.29219/fnr.v67.9374

**Published:** 2023-07-03

**Authors:** Seong-Hoo Park, Jeongjin Park, Minhee Lee, Jinhak Kim, Sangwon Eun, Woojin Jun, Ok-Kyung Kim, Jeongmin Lee

**Affiliations:** 1 Department of Medical Nutrition, Kyung Hee University, Yongin, Republic of Korea; 2 Division of Food and Nutrition and Human Ecology Research Institute, Chonnam National University, Gwangju, Republic of Korea; 3 R&D Division, Daehan Chemtech Co. Ltd. Seoul, Republic of Korea

**Keywords:** obesity, Kaempferia parviflora, lipolysis, adipogenesis, lipogenesis

## Abstract

**Background:**

Obesity occurs when energy intake is excessive compared to energy expenditure, resulting in the excessive storage of triglyceride in adipose tissue.

**Objective:**

The present study aimed to investigate the antiobesity effects of *Kaempferia parviflora* extracts (PF) in high-fat diet (HFD)-induced obese mice and 3T3-L1 adipocytes to demonstrate the lipid mechanisms underlying these effects.

**Design:**

Mice were fed with a normal diet (AIN93G normal diet), HFD (60% HFD), Met (HFD containing metformin 250 mg/kg b.w.), PF50 (HFD containing PF 50 mg/kg b.w.), and PF100 (HFD containing PF 100 mg/kg b.w.) for 12 weeks.

**Results:**

Body weight gain, adipose tissue weight, adipose tissue mass, and size of adipocytes were significantly decreased by PF supplementation in HFD-fed mice. Moreover, PF supplementation suppressed the adipogenesis and lipogenesis pathways and activated the lipolysis and thermogenesis pathways in the adipose tissues of HFD-fed mice.

**Conclusions:**

PF treatment during the differentiation of 3T3-L1 cells suppressed adipogenesis and lipogenesis and PF treatment after differentiation activated lipolysis and thermogenesis. Thus, we suggest that PF is effective for weight loss by directly affecting the lipid metabolism of adipocytes.

## Popular scientific summary

- Supplementation with Kaempferia parviflora extracts significantly reduced body weight gain, adipose tissue weight, and adipose tissue mass in HFD-fed mice.
- Supplementation with Kaempferia parviflora extracts modulated key pathways involved in lipid metabolism, suppressing adipogenesis and lipogenesis while promoting lipolysis and thermogenesis in adipose tissues.

Obesity, a medical condition associated with abnormal fat accumulation, and metabolic syndrome are complexly influenced by diet, environment, low physical activity, and heredity (1). A marked increase in the prevalence of overweight and obesity among children and adolescents was observed from the 1980s to the 1990s, and the obese population has steadily increased since then. Obesity epidemics were first identified in the United States, but over the past few decades, they have become prevalent worldwide. Obesity occurs when energy intake is excessive compared to energy expenditure, resulting in the excessive storage of triglyceride (TG) in adipose tissue. Although high-energy diet intake concurrent with low physical activity plays an important role in the development of obesity, diet-related metabolic changes may be more important (2). Chronic consumption of a high-energy density diet including snacks, chocolates, sugar-sweetened beverages, and fast foods has been associated with an increased prevalence of obesity and metabolic syndrome (3). The increasing consumption of a high-energy density diet has become a serious global health problem (4).

Adipocytes store TG in a state of energy excess and release fatty acids during fasting or times of high energy demand to supply them to other tissues. Thus, adipose tissue is important for maintaining nutritional homeostasis (5). Adipocytes differentiate from multipotent mesenchymal stem cells, and this involves a set of adipogenic transcription factors. After differentiation into mature adipocytes, cell expansion is driven by lipogenesis, which promotes the accumulation of intracellular TG in a nutrient excess state (6). Conversely, lipolysis is induced under physiological conditions when metabolic fuel is low and/or the energy demand is high. Lipolysis generates glycerol and free fatty acids from the enzymatic cleavage of TG by lipases, including adipose TG lipase (ATGL), hormone-sensitive lipase (HSL), and monoacyl-glycerol lipase (6, 7). Therefore, the balance between lipogenesis and lipolysis helps maintain energy homeostasis, and the inhibition of adipogenesis and lipogenesis and stimulation of lipolysis can prevent obesity (5–7).

In this study, we investigated the antiobesity effects of *Kaempferia parviflora*, known as Thai black ginger. *Kaempferia parviflora* belongs to the Zingiberaceae family and has been used in Thai traditional medicine for stomach protection and relief of allergies and fatigue (8, 9). *Kaempferia parviflora* was previously demonstrated to possess various biological activities, such as anticancer and anti-inflammation effects, enhanced endurance capacity effects, and loss of body weight; however, studies on its effects on the lipid metabolism of adipocytes are still lacking (8–10). We confirmed the effect of *Kaempferia parviflora* extracts (PF) on adipogenesis, lipogenesis, and lipolysis in high-fat diet (HFD)-induced obese mice and 3T3-L1 adipocytes to demonstrate the mechanisms involved in the antiobesity effects of *Kaempferia parviflora*.

## Materials and methods

### Preparation of PF

**Fig. 1:**
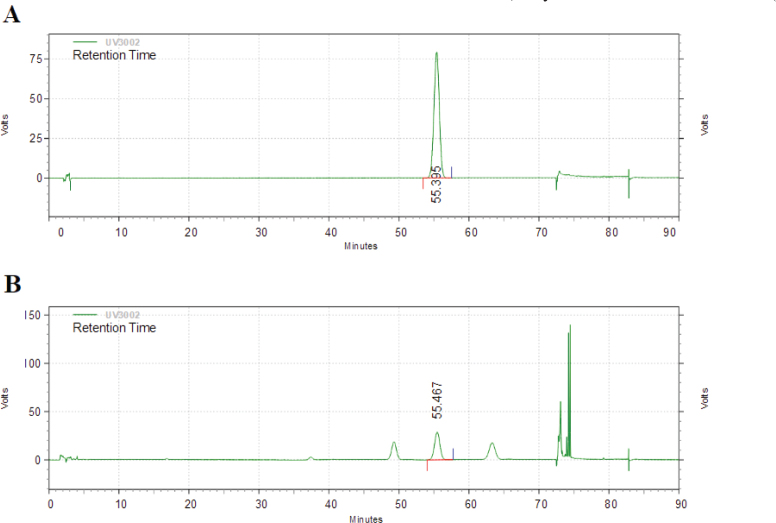
High-performance liquid chromatography analysis of 5,7-dimethoxyflavone in PF. Standard (A) and PF (B).

The standardized rhizome extract of *Kaempferia parviflora* was provided by DAEHAN CHEMTECH Co., Ltd. (Seoul, Korea). The dried rhizome of *Kaempferia parviflora* was extracted in hydrous ethanol. The extracted solution was then filtered, concentrated, and dried to produce dried extracts (SIRTMAX, Tokiwa phytochemical Co., Ltd. Tokyo, Japan; PF). The 5,7-dimethoxyflavone was performed by high-performance liquid chromatography UltiMate 3000 system (Dionex Co., Sunnyvale, California, USA) for standardization. The PF was a standardized more than 4% 5,7-dimethoxyflavone (Fig. 1).

### HFD-fed mouse model

C57BL/6J mice (4-week-old mice, male) were purchased from Saeron Bio (Uiwang, Korea) and housed in cages under controlled conditions (22 ± 2°C, 55% humidity, and a 12:12 h light-dark cycle). Mice were allowed to adapt to conditions for 1 week. Thereafter, they were fed with a normal diet (AIN93G normal diet), HFD (60% HFD), Met (HFD containing metformin 250 mg/kg b.w.), PF50 (HFD containing PF 50 mg/kg b.w.), or PF100 (HFD containing PF 100 mg/kg b.w.) for 15 weeks. Mice were killed and their tissues and blood (by orbital venipuncture) were collected. The experimental protocol was approved by the Institutional Animal Care and Use Committee of Kyung Hee University (KHGASP-22-212). We followed the methods of Yun et al. (11).

### Micro-CT

Mice were examined by whole via abdominal tomography using micro-CT equipment (VIVA CT 80, Scano Medical AG, Switzerland).

### Hematoxylin and eosin staining

Epididymal adipose tissues from mice were fixed with 10% neutral buffered formaldehyde solution and embedded in paraffin. The paraffin blocks were sliced into 5 μm sections, stained with hematoxylin and eosin (H&E), and observed using an optical microscope.

### 3T3-L1 cell culture and treatment

**Fig. 2:**
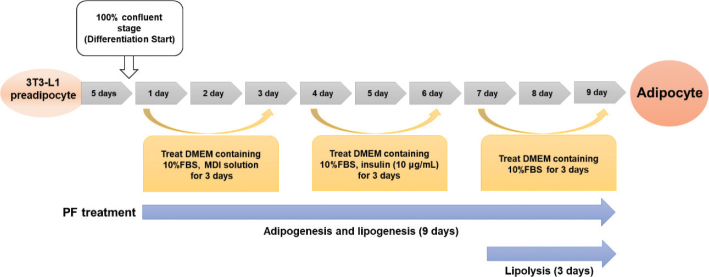
Experimental schematic of differentiation into mature adipocytes and treatment PF.

The 3T3-L1 preadipocyte cell line was purchased from the American Type Culture Collection (Rockville, MD, USA) and cultured under appropriate conditions (95% air, 5% CO_2_, 37°C). Cells were grown in high-glucose DMEM, supplemented with 10% NCS, 1% P/S, 1% L-glutamine, and a 1% sodium pyruvate mixture. Differentiation into mature adipocytes proceeded as shown in the Fig. 6, according to methods described previously (Fig. 2) (12).

### Western blot

Proteins were extracted from the epididymal adipose tissues and brown adipose tissues (BAT) of mice and cells were analyzed for the expressions of p-MAPK (Erk1/2), mitogen-activated protein kinase (MAPK) (Erk1/2), p-CREB, cAMP response element-binding protein (CREB), element-binding protein1 (SREBP1), peroxisome proliferator-activated receptor γ (PPAR-γ), CCAAT/enhancer binding proteins (C/EBPα), citrate synthase, p-ACL, ATP-citrate lyase (ACL), p-ACC, acetyl-CoA carboxylase (ACC), fatty acid synthase (FAS), lipoprotein lipase (LPL), ATGL, protein kinase A (PKA), p-HSL, HSL, p-AMPK, AMP-activated protein kinase (AMPK), uncoupling protein-1 (UCP-1), carnitine palmitoyltransferase (CPT1)A, and β-actin according to methods described previously (11, 12).

### Oil red O staining

On the 9th day of differentiation, cells were washed with DPBS, fixed with 10% formalin, and the plate dried with 60% isopropanol. Lipid sphere staining was performed for 2 h with the Oil Red O working solution and then washed four times with distilled water and photographed. To measure the accumulation of lipids, 100% isopropanol was added to the well-dried plate to elute Oil red O dye.

### Measurement of cAMP levels

cAMP levels (cAMP ELISA kit, Cell biolabs Inc., San Diego, CA, USA) were measured in the white adipose tissue (WAT) of mice and in differentiated 3T3-L1 cells by ELISA. The experiment was conducted according to each manufacturer’s manual.

### Statistical analysis

All data are expressed as the mean ± standard deviation (SD). Significant differences were determined using one-way analysis of variance (ANOVA) and Duncan’s multiple range test (SPSS PASW Statistic v.23.0, SPSS Inc., Chicago, IL, USA). Statistical significance was determined at *P* < 0.05.

## Results

### Effects of Kaempferia parviflora on the body and organ weights of HFD-fed mice

The weight gain, food efficiency ratio, and tissue weights of the liver and adipose tissues of mice in HFD groups were significantly increased compared to those from the normal diet group, as expected. However, dietary supplementation with metformin (Met) and PF decreased the weight gain, and tissue weights of the liver and adipose tissues of mice compared to the HFD-fed mice (Table 1) (*P* < 0.05). These findings suggest that dietary supplementation with PF can suppress weight gain during HFD intake.

**Table 1:** Effects of PF on the body and organ weights of high-fat diet-fed mice

	Normal diet	HFD	Met	PF50	PF100
Weight gain (g)	10.16 ± 1.24^d^	24.78 ± 1.92^a^	16.75 ± 1.22^c^	20.81 ± 2.29^b^	19.37 ± 1.12^d^
Food efficiency ratio	5.09 ± 1.81^c^	8.45 ± 0.66^a^	6.87 ± 0.50^b^	8.47 ± 0.90^a^	8.31 ± 0.76^a^
Tissue weights (g)					
Liver	1.46 ± 0.15^c^	2.10 ± 0.20^a^	1.81 ± 0.22^b^	1.95 ± 0.20^ab^	1.85 ± 0.14^b^
Spleen	0.42 ± 0.03^NS^	0.45 ± 0.04	0.43 ± 0.03	0.43 ± 0.03	0.44 ± 0.03
Kidney	0.22 ± 0.01^NS^	0.22 ± 0.01	0.22 ± 0.01	0.22 ± 0.01	0.22 ± 0.01
Subcutaneous fat	0.91 ± 0.14^e^	2.70 ± 0.27^a^	1.58 ± 0.17^d^	2.29 ± 0.22^b^	1.88 ± 0.11^c^
Visceral fat	1.43 ± 0.24^d^	3.91 ± 0.29^a^	2.70 ± 0.36^c^	3.43 ± 0.25^b^	3.01 ± 0.32^c^
Total white adipose tissue	2.34 ± 0.35^e^	6.61 ± 0.39^a^	4.29 ± 0.26^d^	5.75 ± 0.35^b^	4.89 ± 0.35^c^
Brown adipose tissue	0.20 ± 0.02^d^	0.30 ± 0.03^a^	0.22 ± 0.01^c^	0.25 ± 0.01^b^	0.23 ± 0.02^bc^

Normal diet, HFD (60% high-fat diet), Met (HFD containing metformin 250 mg/kg b.w.), PF50 (HFD containing PF 50 mg/kg b.w.), and PF100 (HFD containing PF 100 mg/kg b.w.). Values are presented as the mean ± standard deviation (*n* = 8). Different letters (a > b > c > d > e) indicate significant differences with *P* < 0.05, as determined using Duncan’s multiple range test.

### Effects of Kaempferia parviflora on adipose mass and adipocyte size in HFD-fed mice

**Fig. 3:**
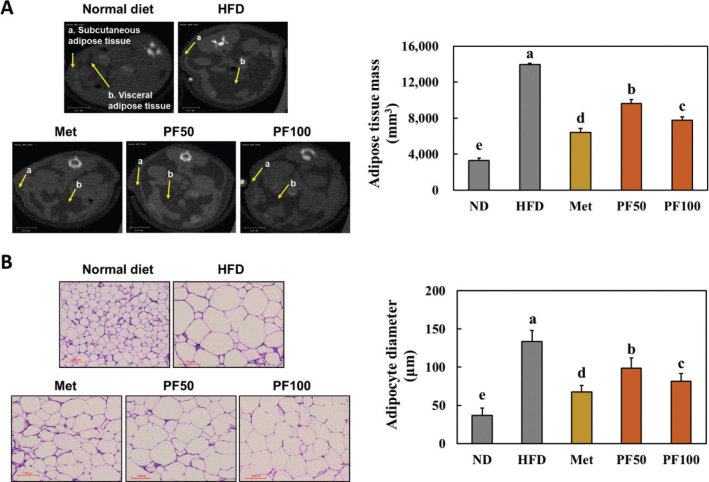
Effects of PF on adipose tissue mass (A) and size of adipocytes (B) in high-fat diet-fed mice. Normal diet, HFD (60% high-fat diet), Met (HFD containing metformin 250 mg/kg b.w.), PF50 (HFD containing PF 50 mg/kg b.w.), and PF100 (HFD containing PF 100 mg/kg b.w.). Values are presented as the mean ± standard deviation (*n* = 8). Different letters (a > b > c > d > e) indicate significant differences with *P* < 0.05, as determined using Duncan’s multiple range test.

We examined the distribution of subcutaneous and visceral adipose tissues in the abdomen and total adipose tissue mass using micro-CT. We found that HFD-fed mice had a significant increase in visceral adipose tissues and total adipose tissue mass (Fig. 3A) compared to that in the normal diet-fed mice. In addition, the adipocyte size was significantly increased in HFD-fed mice compared to that in the normal diet-fed mice (Fig. 3B). However, dietary supplementation with Met and PF significantly decreased the adipose tissue mass and adipocyte size in HFD-fed mice compared to that in the HFD group (Fig. 3) (*P* < 0.05). Thus, we suggest that dietary supplementation of PF can suppress body weight gain by decreasing adipose tissue mass and adipocyte size.

### Effect of Kaempferia parviflora on lipid metabolism in the adipose tissues of HFD-fed mice

**Fig. 4:**
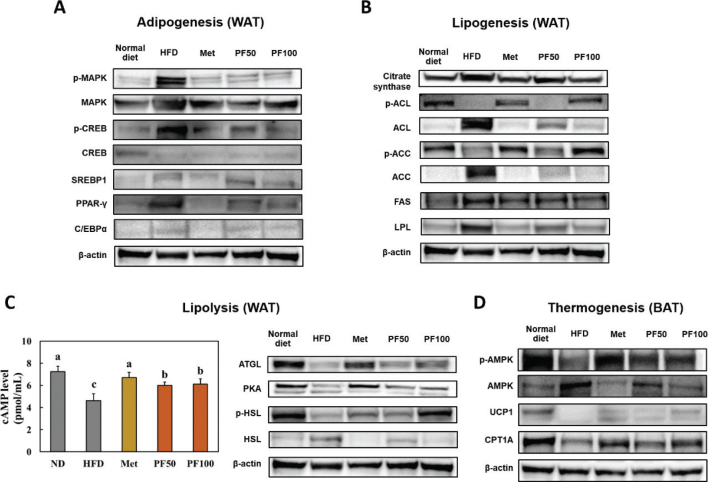
Effects of PF on the adipogenesis (A), lipogenesis (B), and lipolysis (C) pathways in white adipose tissue and the thermogenesis pathways in brown adipose tissue (D) from high-fat diet-fed mice. Normal diet, HFD (60% high-fat diet), Met (HFD containing metformin 250 mg/kg b.w.), PF50 (HFD containing PF 50 mg/kg b.w.), and PF100 (HFD containing PF 100 mg/kg b.w.). Values are presented as the mean ± standard deviation (*n* = 8). Different letters (a > b > c) indicate significant differences with *P* < 0.05, as determined using Duncan’s multiple range test.

We examined lipid metabolism including adipogenesis, lipogenesis, lipolysis, and thermogenesis in adipose tissues using western blotting. The expression levels of proteins involved in the adipogenesis pathway, phosphorylated MAPK and CREB, and adipogenic transcription factors, were significantly increased in WAT from the HFD group compared to those from the normal diet group (Supplementary Fig. S1, Fig. 4A). Moreover, the expression levels of proteins involved in the lipogenesis pathway, citrate synthase, dephosphorylated ACL and ACC, FAS, and LPL, were significantly increased in the WAT from HFD group compared to those from the normal diet group (Supplementary Fig. S1, Fig. 4B). Moreover, lipolysis-related factors, cAMP level, ATGL, PKA, and phosphorylated HSL, were significantly decreased in the WAT from the HFD group compared to those from the normal diet group (Supplementary Fig. S1, Fig. 4C). Thermogenesis-related proteins including phosphorylated AMPK, UCP-1, and CPT1 in the BAT from HFD-induced obese mice were significantly decreased compared to levels in the normal diet-fed mice. However, adipogenesis and lipogenesis factors were significantly decreased, and lipolysis and thermogenesis factors were significantly increased in PF-supplemented groups compared to those in the HFD group (Supplementary Fig. S1, Fig. 4) (*P* < 0.05).

### Effect of Kaempferia parviflora on adipogenesis and lipogenesis in 3T3-L1 cells during differentiation

**Fig. 5:**
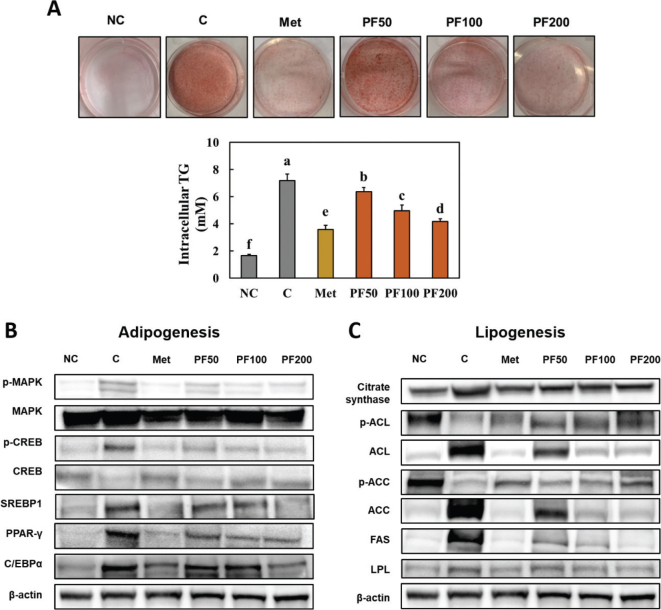
Effects of PF on the intracellular TG (A) and adipogenesis (B) and lipogenesis (C) pathways in 3T3-L1 cells during differentiation. NC (Normal control; undifferentiated 3T3-L1 cells), C (Control; differentiated 3T3-L1 cells), Met (3T3-L1 cells with metformin treatment at 1 mM during differentiation), PF50 (3T3-L1 cells with PF treatment at 50 μg/mL during differentiation), PF100 (3T3-L1 cells with PF treatment at 100 μg/mL during differentiation), and PF200 (3T3-L1 cells with PF treatment at 200 μg/mL during differentiation). Different letters (a > b > c > d > e > f) indicate significant differences with *P* < 0.05, as determined using Duncan’s multiple range test.

We found that PF treatment in 3T3-L1 cells during differentiation suppressed intracellular TG in a dose-dependent manner, compared to the control 3T3-L1 cells (Fig. 5A). We measured the expression levels of proteins in the adipogenesis and lipogenesis pathways in PF-treated 3T3-L1 cells during differentiation and found PF treatment suppressed their expression levels compared to the control 3T3-L1 cells (Supplementary Fig. S2, Fig. 5B and C). These results indicated that PF can directly affect the adipogenesis and lipogenesis pathways in adipocytes.

### Effect of Kaempferia parviflora on lipolysis and thermogenesis in 3T3-L1 cells after differentiation

**Fig. 6:**
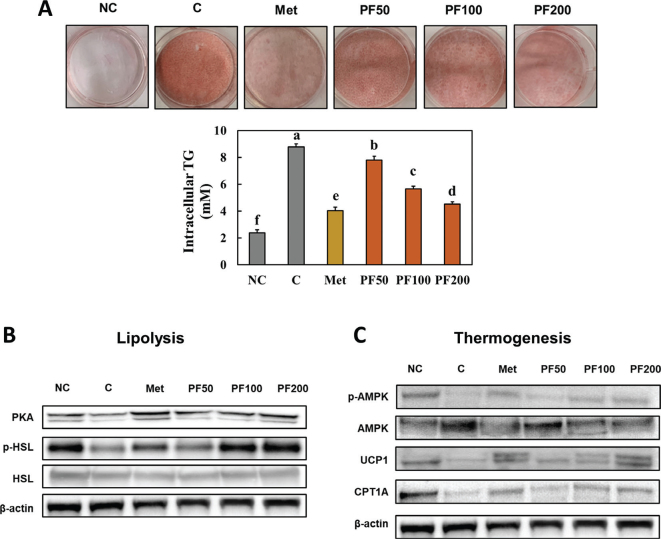
Effects of PF on intracellular TG (A) and the lipolysis (B) and thermogenesis (C) pathways in 3T3-L1 cells after differentiation. NC (Normal control; undifferentiated 3T3-L1 cells), C (Control; differentiated 3T3-L1 cells), Met (3T3-L1 cells with metformin treatment at 1 mM after differentiation), PF50 (3T3-L1 cells with PF treatment at 50 μg/mL after differentiation), PF100 (3T3-L1 cells with PF treatment at 100 μg/mL after differentiation), and PF200 (3T3-L1 cells with PF treatment at 200 μg/mL after differentiation). Different letters (a > b > c > d > e > f) indicate significant differences with *P* < 0.05, as determined using Duncan’s multiple range test.

We examined the intracellular TG and lipolysis and thermogenesis pathways in 3T3-L1 cells after differentiation to investigate the effects of PF on lipolysis in differentiated adipocytes. PF treatment suppressed intracellular TG (Fig. 6A) and stimulated the lipolysis and thermogenesis pathways in the differentiated 3T3-L1 cells compared to the control 3T3-L1 cells (Supplementary Fig. S2, Fig. 6B and C). Therefore, PF treatment can directly decrease TG by stimulating lipolysis and thermogenesis in adipocytes.

## Discussion

In the present study, we investigated whether supplementation of a diet with PF could reduce the body weight as well as liver weight, subcutaneous fat fad, visceral fat fad, adipose tissue mass, and size of adipocytes in HFD-fed mice. Yoshino et al. demonstrated that the administration of PF promoted energy metabolism in the BAT by upregulating UCP1 protein (13). The present study demonstrated the effect of *Kaempferia parviflora* on antiobesity with a mechanism related to lipid metabolism by examining the WAT and BAT of HFD-fed mice. We used Met, a drug for the treatment of type 2 diabetes, to promote weight loss via the activation of AMPK, as a positive control (14). Reusch et al. reported that CREB plays a role in the differentiation of 3T3-L1 preadipocytes to adipocytes, and previous studies have indicated that MAPKs are involved in adipocyte differentiation (15). MAPK and CREB stimulate sterol regulatory SREBP1, PPARγ, and C/EBPs for adipogenesis (15, 16). We showed that HFD induced the expressions of phosphorylated MAPK and CREB, SREBP1, PPARγ, and C/EBPα in the WAT of mice and differentiated 3T3-L1 adipocytes. Interestingly, dietary supplementation of HFD-fed mice with PF and treatment of 3T3-L1 adipocytes with PF showed a decreased protein expression of adipogenesis factors, indicating PF can inhibit adipocyte differentiation.

Excess energy can be stored in the fat, particularly adipose tissue, by converting acetyl-CoA to TG, which is a metabolic process termed *de novo* lipogenesis. In addition, excess fatty acids in the blood are internalized by adipocytes and stored in the fat (17, 18). We measured the expressions of lipogenesis-related enzymes, including ACL, ACC, and FAS, in the WAT of HFD-fed mice and 3T3-L1 adipocytes. We showed that dietary supplementation of HFD-fed mice with PF and treatment of 3T3-L1 adipocytes with PF decreased the protein expressions of lipogenesis-related enzymes. Therefore, these findings suggest that PF has potent antiobesity activity by reducing fat accumulation via the inhibition of adipogenesis and lipogenesis.

It is well known that excess fat accumulation is caused by a chronic imbalance between energy intake and energy expenditure. Thus, drugs for obesity therapy reduce body weight by mechanisms related to a decrease in the intestinal absorption of food substrates, decreased appetite, or increase in energy expenditure. Among these mechanisms, increasing energy expenditure is important for long-term clinical efficacy targeting obesity (17–21). Fatty acids are produced by the catabolism of TG induced by the activation of lipase in adipocytes, which then generates ATP by β-oxidation and promotes the TCA cycle (21). We found PF stimulated the protein expression of ATGL and phosphorylation of HSL in HFD-fed mice and 3T3-L1 adipocytes. Moreover, PF stimulated the protein expression of phosphorylated AMPK, CPT1, and UCP1 in HFD-fed mice and 3T3-L1 adipocytes. The phosphorylation of AMPK can upregulate CPT1 and UCP1, key molecules for thermogenesis. Therefore, we suggest that PF can stimulate energy expenditure by stimulating lipogenesis and thermogenesis.

A study by Shimada et al. showed that ethyl acetate extracts of *Kaempferia parviflora* had preventive effects on symptoms related to insulin resistance, hypertension, and fatty liver in an obese Type II diabetes animal model (22). In addition, Akase et al. also found that extracts of *Kaempferia parviflora* suppressed fat accumulation, hyperinsulinemia, glucose intolerance, insulin resistance, and hypertension in an obese Type II diabetes animal model (23). In this study, we investigated the antiobesity effects of *Kaempferia parviflora* on lipid metabolism in a diet-induced obese model. These previous studies and our findings demonstrate that *Kaempferia parviflora* is effective for weight loss by affecting lipid metabolism. *Kaempferia parviflora* contains major phytochemicals including 5,7-dimethoxyflavone and 5,7,4′-trimethoxyflavone (24), and the further study each phytochemical in *Kaempferia parviflora* is needed to investigate the molecular mechanisms in more detail.

In the present study, we investigated the antiobesity effects of *Kaempferia parviflora* by observing its effects on lipid metabolism including adipogenesis, lipogenesis, lipolysis, and thermogenesis. Our findings showed that *Kaempferia parviflora* inhibited the adipogenesis and lipogenesis pathways and activated the lipolysis and thermogenesis pathways in HFD-fed mice and 3T3-L1 adipocytes. This study provides scientific evidence for the weight loss effects of *Kaempferia parviflora* and explains the underlying mechanisms involved.

## Conflict of interest and funding

The authors have no conflicts of interest to declare. No funding was received.
