# Supplementary material for: Antiobesity effect of Kaempferia parviflora accompanied by inhibition of lipogenesis and stimulation of lipolysis
Source: Food Nutr Res. 2023 Jul 3;67:10.29219/fnr.v67.9374. doi: 10.29219/fnr.v67.9374 (PMC10335092; doi:10.29219/fnr.v67.9374)
Supplement: Supplementary file 1 [file FNR-67-9374-s001.docx]

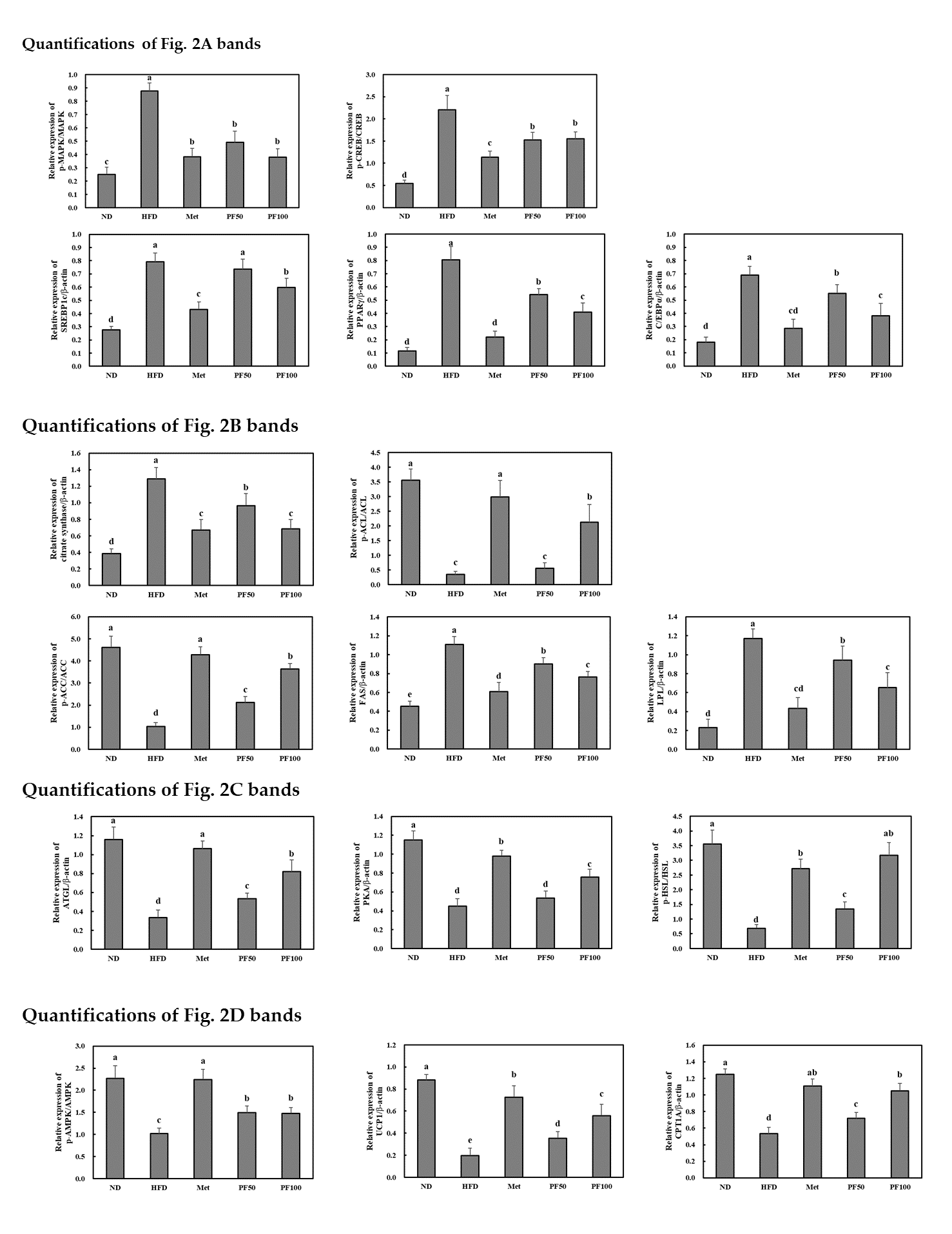


**Sup 1. Quantification of the western blot bands relative expression in Figure 2.**

**
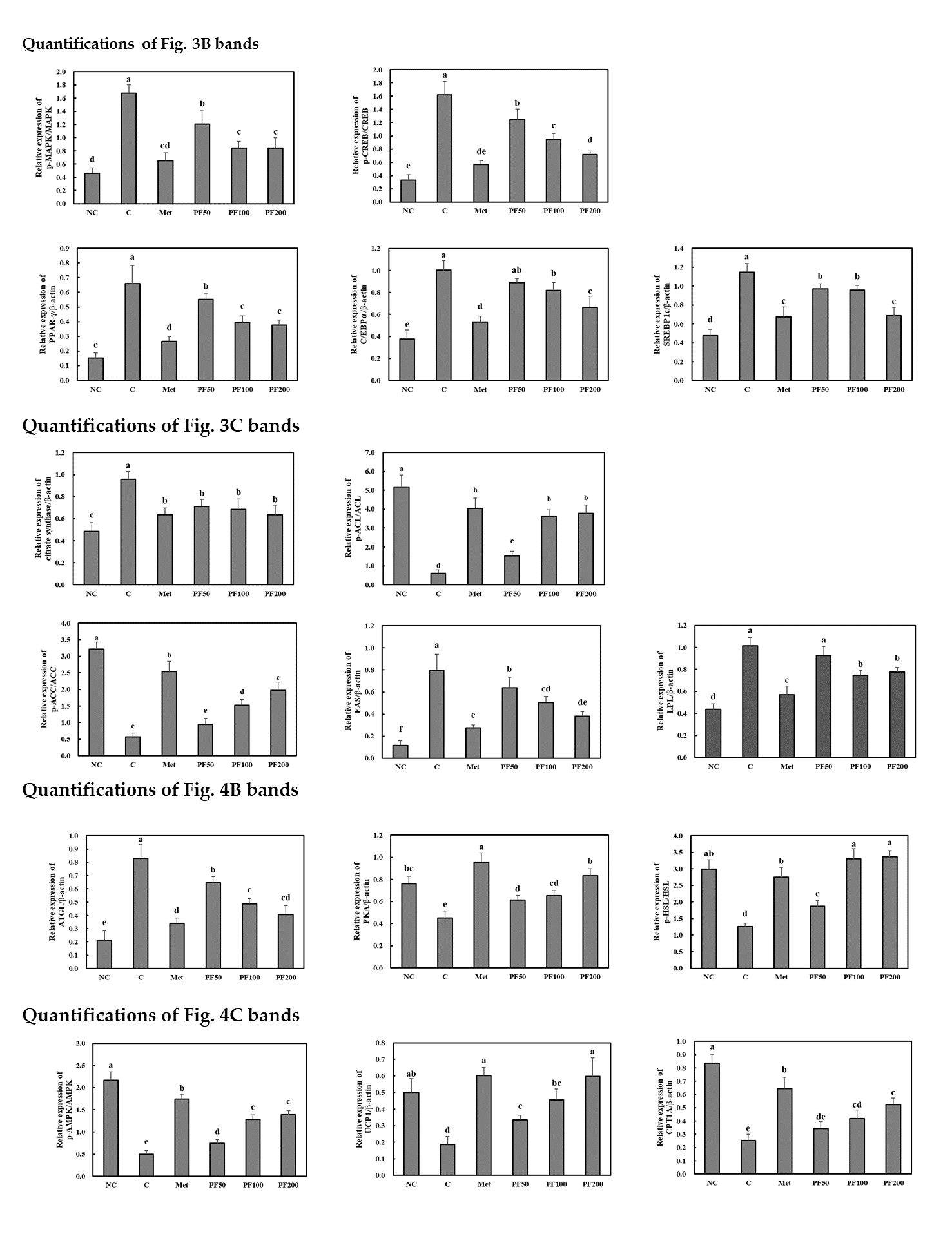
**

**Sup 2. Quantification of the western blot bands relative expression in Figure 3 and 4.**
